# Differentiation ability of hematopoietic stem cells and mesenchymal stem cells isolated from human peripheral blood

**DOI:** 10.3389/fcell.2024.1450543

**Published:** 2024-12-18

**Authors:** Echambadi Loganathan Samundeshwari, Surekha Kattaru, Sireesha Kodavala, Chodimella Chandrasekhar, Potukuchi Venkata Gurunadha Krishna Sarma

**Affiliations:** ^1^ Department of Biotechnology, Sri Venkateswara Institute of Medical Sciences, Tirupati, Andhra Pradesh, India; ^2^ Department of Hematology, Sri Venkateswara Institute of Medical Sciences, Tirupati, Andhra Pradesh, India

**Keywords:** peripheral blood stem cells, human hematopoietic stem cells, mesenchymal stem cells, differentiation ability, regenerative medicine

## Abstract

Human hematopoietic stem cells (HSCs) and mesenchymal stem cells (MSCs) are the major stem cells of the bone marrow and are usually isolated from the peripheral blood. In the present study, we isolated these stem cells by an apheresis method from a donor who was administered granulocyte colony-stimulating factor (G-CSF). *In vitro* propagation of these stem cells showed a plastic-adherence property expressing CD73 and CD105 surface markers, which is a characteristic feature of MSCs. HSCs are non-adherent cells growing as a suspension culture, expressing CD150, CD133, CD34, and CD45 on their surface, which regulate the quiescence nature, and they derive energy from anaerobic glycolysis. The HSCs grow slowly compared to MSCs, are more viable, and survive for long periods under *in vitro* conditions, which are due to the expression of telomerase, BCL2, and Notch1 genes. The poor viability of MSCs in the culture due to the prominent expression of apoptotic genes BAX, caspase-3, and caspase-9 leads to rapid apoptosis. This was evident even in cells (astrocytes, osteocytes, and beta cells of the islets of Langerhans) differentiated from HSCs and MSCs, thus highlighting the importance of HSCs, the naive stem cells, in regeneration of tissues.

## Graphical Abstract

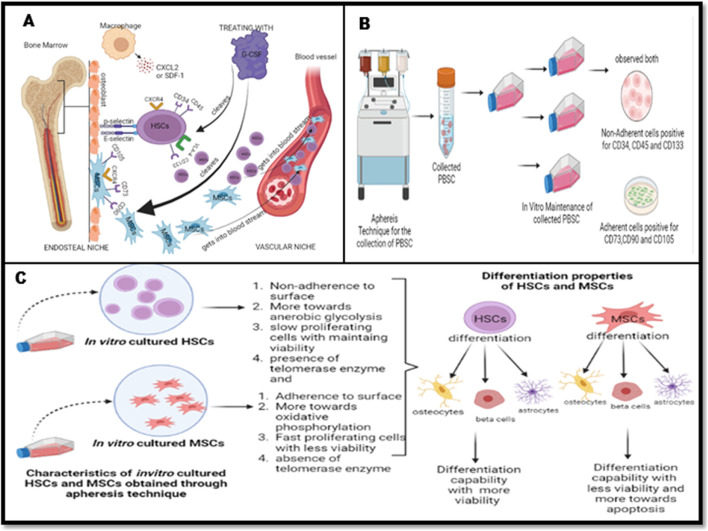

## Introduction

Hematopoietic stem cells (HSCs) and mesenchymal stem cells (MSCs) are the major stem cells of the bone marrow (BM) (Battiwalla and Hematti, 2009). The HSCs are bound to the endosteum through a series of proteins, i.e., N-cadherin, c-Kit, CXCR4, very late antigen-4 (VLA-4), E-selectin ligand-1 (ESL-1), and P-selectin glycoprotein ligand-1 (PSGL-1), secreted by the HSCs, while MSCs are directly attached to the cells of the bone marrow (Kulkarni and Kale, 2020). Recent findings highlight that the balance between quiescence, self-renewal, and differentiation in BM is crucial for HSC survival and preparation for differentiation into hematopoietic stem/progenitor cells (HSPCs) (Seita and Weissman, 2010). Furthermore, HSCs are classified into short-term HSCs (ST-HSCs), which exhibit higher differentiation potential, and long-term HSCs (LT-HSCs), which have extensive self-renewal capabilities. HSC differentiation into blood lineages such as red blood cells, platelets, and lymphoid/myeloid cells is regulated by cytokines like granulocyte colony-stimulating factor (G-CSF) and thrombopoietin (TPO); transcription factors such as PU.1 and GATA-1; and key signaling pathways, including JAK-STAT, MAPK, and PI3K/AKT (Mann et al., 2022; Raghav and Gangenahalli, 2021). Additionally, the receptor tyrosine kinase c-Kit, through its interaction with stem cell factor (SCF) and downstream pathways like STAT-3, plays a pivotal role in HSC proliferation, with studies showing that inhibition of protein tyrosine phosphatases (SHP-1 and SHP-2) enhances c-Kit-mediated growth (Mann et al., 2022; Raghav et al., 2018a; Raghav et al., 2018b; Raghav and Gangenahalli, 2018; Edling and Hallberg, 2007). Understanding these molecular mechanisms is critical for improving therapies in bone marrow transplantation and hematopoietic disorders. MSCs, with their regenerative potential and multi-lineage differentiation capacity, also hold promise in therapeutic applications. MSC-based biomaterials, such as nanocomposites and exosomes, represent advanced strategies in regenerative medicine and tissue engineering, enhancing tissue repair, immune regulation, and differentiation, which make MSCs key tools for next-generation therapies (Raghav et al., 2022; Rawat et al., 2021; Raghav and Mann, 2021).

These HSCs and MSCs were mobilized into the blood stream by the action of G-CSF and interleukin-3, and these stimulate neutrophils to secrete proteases, which neutralizes the interaction of the HSC with its niche and enables the HSCs and MSCs to move into peripheral blood. Collectively, these cells are referred as peripheral blood stem cells (PBSCs) (Bernitz et al., 2017). Since 1986, these peripheral blood stem cells have been directly used 100% in the autologous and approximately 75% in allogeneic transplantation procedures (Körbling and Freireich, 2011). The conditions for use of peripheral blood stem cells (contains HSCs and MSCs) in regenerative medicine require pure culture; *ex vivo* expansion, with maintaining its viability; ability of differentiation; self-renewal; and the presence of telomerase activity, which are essential for successful transplantation (Seita and Weissman, 2010).

Experimentally, these PBSCs were mobilized by administering G-CSF to the donor/patients for 3 consecutive days, followed by harvesting these cells through an apheresis technique using a cell separator. Such apheretically separated PBSCs are enumerated by using the expression of surface markers CD34/CD133/CD45, with HSCs showing positivity to these markers, while MSCs show positivity toward CD73/CD105 surface markers. For over 60 years, transplantation of HSCs has been the major curative therapy for several genetic and hematological disorders (Bernitz et al., 2017). Almost in 1963, Till and McCulloch described a single-progenitor cell type in the bone marrow which expands clonally and gives rise to all lineages of hematopoietic cells. This research represented the first characterization of the HSCs. These HSCs exhibit self-renewal ability and potential to lineage-specific differentiation (Körbling and Freireich, 2011). The biology of HSCs became more interesting with the cloning and sequencing of CD34, CD133, and CD45 genes, which expressed on the surface of HSCs and also regulate the functions of HSCs. They are the major markers for the isolation of pure HSCs from peripheral blood. The prominent expression of CD150 on HSCs explains that the isolated HSCs have a quiescent nature in the bone marrow endosteal niche. The self-renewal and long term survival characteristic of HSCs is its ability to elongate its telomere sequence with the active telomerase enzyme, which explains the lifelong replenishing ability of blood cells by HSCs (Sekulovic et al., 2011).

Pure HSCs are the prerequisite for any transplantation studies or gene therapy, followed by the transplantation of such genetically altered HSCs. Such studies were carried out three decades back in patients suffering from severe combined immune deficiency (SCID) (Cavazzana-Calvo and Fischer, 2007). Pure HSCs of such patients, who had the defective adenosine deaminase (ADA) gene responsible for SCID, were corrected and transplanted into the same patient, resulting in enhanced immunity in the SCID patient. The importance of HSCs was re-established when induced pluripotent stem cells were made to differentiate into HSCs, and such HSCs were directly used in regenerative medicine (Esposito, 2016). Although MSCs are a promising cell source for use in tissue regeneration and their potential therapeutic application is already under investigation, several studies have demonstrated that the long-term culture of MSCs results in continuous changes to the cells, including the proliferation rate, cell size, and different differentiation potentials (Kim and Cho, 2013). Furthermore, these MSCs undergo rapid apoptosis, and these problems have hindered the expansion of MSCs for therapeutic use, causing a major bottleneck in clinical applications (Mastrolia et al., 2019). In this backdrop, the present study aimed to isolate and characterize HSCs and MSCs and their ability for lineage-specific differentiation and describe the importance of HSCs and MSCs in regenerative medicine.

## Methodology

### Antibodies

The following antibodies (Abs) were used for flow cytometry: FITC anti-human CD34, FITC anti-human CD45, PE anti-human CD73, PE anti-human Ki67, FITC anti-human CD105, and PE anti-human CD133. All the antibodies were procured from BioLegend and were also used for the immunofluorescence staining of cells.

### Isolation and maintenance of PBSCs

Bone marrow stem cells were mobilized to peripheral blood with 5 μg/kg/day G-CSF for up to 3 consecutive days in a donor, and the mobilized stem cells were separated by using an Rvy kit fitted to an automated blood cell separator system. The obtained cells were cultured in Dulbecco’s modified Eagle’s medium (DMEM) containing 10% FBS and maintained at 37°C with 5% CO_2_ and 95% humidity. The protocol was approved by the Institutional Ethical Committee (IEC; Nos: 31/06/2006 and 419/27-01-2015) of SVIMS University, and research work was carried out in accordance with the guidelines of the World Medical Association and Declaration of Helsinki, with written consent obtained from the individual prior to the initiation of experiments (Sarma and Subramanyam, 2008).

### Characterization of cultured PBSCs

#### Characterization of HSCs by immunocytochemistry, fluorescence microscopy, and flow cytometry

*In vitro* cultured peripheral blood stem cells were morphologically observed by staining with 3% Giemsa, and for immunocytochemistry (ICC), the cells were incubated with monoclonal mouse anti-human CD34 as the primary antibody and rabbit anti-mouse IgG-HRP conjugate as the secondary antibody. Flow cytometry and immunofluorescence analysis for the identification of CD34, CD133, and CD45 surface markers were performed using the antibodies mentioned above following the ISHAGE guidelines. Real-time quantitative polymerase chain reaction (qRT-PCR) was performed using the Applied Biosystems ABI 7300 System using CD34, CD45, CD133, and CD150 gene-specific primers with SYBR Select Master Mix (Gibco, Invitrogen). Genes were taken from a human genome database and were custom-designed using Primer Express Software (Applied Biosystems, United States) (Sutherland et al., 1996).

#### Characterization of MSCs by ICC, fluorescence microscopy, and flow cytometry

*In vitro* cultured peripheral blood stem cells were morphologically observed by staining with 3% Giemsa, and for ICC, the cells were incubated with monoclonal mouse anti-human CD73 as the primary antibody and rabbit anti-mouse IgG-HRP conjugate as the secondary antibody. Flow cytometry and immunofluorescence analysis for the identification of CD73 and CD105 surface markers were performed using the antibodies mentioned above following the ISHAGE guidelines. qRT-PCR was performed using the Applied Biosystems ABI 7300 System using CD73 and CD105 gene-specific primers with SYBR Select Master Mix (Gibco, Invitrogen). Genes were taken from the human genome database and were custom-designed using Primer Express Software (Applied Biosystems, United States) (Baghaei et al., 2017).

#### Proliferation and viability status of HSCs and MSCs

HSCs and MSCs were assed for proliferation by counting the cells using a cytometer, and the viability was assessed by the MTT assay for 24 h and 48 h. Ki-67 staining was performed by fixing HSCs and MSCs on a sterile glass slide with 4% paraformaldehyde, incubating with 300 nM PE anti-human Ki-67 antibody, and observing under a fluorescence microscope. Flow cytometry for Ki-67 was performed (Kumar P. et al., 2018; Graefe et al., 2019).

#### TRAP assay for HSCs and MSCs

Telomerase enzyme activity in HSCs and MSCs was studied by preparing a protein lysate from 1 × 10^6^ cells with a lysis buffer and incubating on ice for 30 min. The protein lysate was transferred to a fresh tube, and the reaction was carried out in two steps: step 1—0.1 mL TRAP reaction containing 5 µL 5 × TRAP buffer, 1 µL dNTP (50 mM), 1 µL TS primer, 0.5 µL Taq-DNA polymerase, and 25 µL H_2_O. The protein lysate was added, and these components were mixed gently and incubated for 30 min at 23°C, followed by 90°C for 3 min in a thermocycler; step 2—to each tube, 1 µL ACX primer, 1 µL NT primer, and 1 µL TSNT primer were added (Table 1), and the telomere repeats were amplified by 30-s denaturation at 94°C, 30-s annealing at 50°C, and 1.5-min polymerization at 72°C for 27 cycles. Furthermore, 10% polyacrylamide gel was used to run TRAP products with 0.5 × TBE as a tank buffer containing ethidium bromide, and the bands were observed under UV in an imaging system (Ray, 2019; Banerjee and Jagadeesh, 2009).

**TABLE 1:** TRAP assay primers and conditions.

Gene	Primer 5′→3′	Condition
TS primer	AATCCGTCGAGCAGAGTT	Denaturation 30 s at 94°C Annealing 30 s at 50°C Polymerization 1.5 min at 72°C
ACX primer	GCG​CGG​CTT​ACC​CTT​ACC​CTT​ACC​CTA​ACC	
NT primer	ATGGCTTCTCGGCCTTTT	
TSNT primer	5′AATCCGTCGAGCAGAGTT AAAAGGCCGAGAAGCGAT-3′	

#### Analysis of enzyme activity in HSCs and MSCs

Cell lysates were prepared by using a lysis buffer (50 mM Tris, 150 mM NaCl, 100 mM EDTA, and 0.5% Triton × 100) by incubating at 37°C for 1 h, followed by centrifugation at 600 rpm for 2 min at 4°C. The supernatant was collected and again centrifuged at 12,000 rpm for 1 h. Thus, the obtained cytosolic fractions were used for enzymatic analysis, and the enzyme assays such as glucokinase (GK), lactate dehydrogenase (LDH), pyruvate kinase (PK), pyruvate dehydrogenase kinase (PDHK), aldehyde dehydrogenase-2 (ALDH2), isocitrate dehydrogenase (IDH), and serine/threonine protein kinase (STPK) were done according to standard procedures (Srikanth et al., 2015; Sunitha et al., 2016a).

#### Quantification of gene expression analysis in HSCs and MSCs

Total RNA was extracted from HSCs and MSCs using the MEDOX-Easy Spin Column Total RNA Miniprep Super Kit. Total RNA (100 ng) in a 40 μL or 100 μL reaction was reverse-transcribed to cDNA using the High-Capacity cDNA Reverse Transcription Kit (Applied Biosystems) primed in the presence of random primers (1 μg). The reaction tube contained MultiScribe reverse transcriptase, 10 × RT buffers, 100 mM dNTP mix, and 10 × random primers, along with the above isolated RNA templates. Reverse transcription was performed in a thermal cycler as per the manufacturer’s protocol. Then, the obtained cDNA was used directly for quantitative PCR. qRT-PCR was performed using the Applied Biosystems ABI 7300 System for the genes HIF1α, IDH, STPK, ALDH, Notch1, cyclin D, BAX, BCL2, caspase-3, caspase-9, telomerase, and GAPDH with gene-specific primers and SYBR Select Master Mix (Gibco, Invitrogen) under the mentioned conditions (Table 2). The results were analyzed by the 2^(−ΔΔct)^ method (Srikanth et al., 2015; Sunitha et al., 2016a; He et al., 2018).

**TABLE 2:** Quantitative RT-PCR primer details.

S. No.	Gene	Forward primer 5′→3′	Reverse primer 5′→3′	Tm°C
1	CD34	CTC​CAG​AAA​CGG​CCA​TTC​AG	CCC​ACC​TAG​CCG​AGT​CAC​AA	59
2	CD45	TCC​AAG​AGG​AAA​GAC​TCT​CGA​ACT	GCA​GGA​AGC​TGC​TCC​ACA​CT	59
3	CD133	ATC​TGC​AGT​GGA​TCG​AGT​TCT​CT	AGCGGTGGCCACAGGTT	59
4	CD150	CTC​CTT​GAC​CTT​CGT​GCT​CTT	GCC​CAC​CTG​TTC​CGT​AGC​T	59
5	CD73	CTG​GGA​CAT​TCG​GGT​TTT​GA	CGT​CCA​CAC​ACC​CCT​CAC​TTT​C	59
6	CD90	GGACTGCCGCCATGAGAA	CTC​ACG​GGT​CAG​GCT​GAA​CT	59
7	CD105	GACCCGCGCTTCAGCTT	TGCCGGTTTTGGGTATGG	59
8	HIF1α	GAA​AGC​GCA​AGT​CCT​CAA​AG	TGG​GTA​GGA​GAT​GGA​GAT​GC	59
9	NOTCH	ACC​CAT​GGT​ACC​AAT​CAT​GAA​TC	TGG​AGG​GAC​CAA​GAA​CTT​GTA​TAA​C	59
10	STPK	GAG​GGT​ACC​TAC​GGC​ATT​GTG	CTT​CAG​TGC​GAC​AAT​CTC​ATC​TG	59
11	ALDH	CCG​GCT​GGG​CTG​ATA​AGT​AC	GGC​GTG​TGT​AGC​TGA​AGA​AGT​CT	59
12	IDH	TGC​TGA​GTT​TGC​TTT​GAG​TAT​G	CGC​ATG​ATG​TTG​GCT​TTG​TG	59
13	TELOMERASE	CCTCACCCACGCGAAAAC	CCGCGGTTGAAGGTGAGA	59
14	CYCLIN D	TGC​ATG​TTC​GTG​GCC​TCT​AA	CGG​TGT​AGA​TGC​ACA​GCT​TCT​C	59
15	BAX	TGG​CAG​CTG​ACA​TGT​TTT​CTG​AC	TCA​CCC​AAC​CAC​CCT​GGT​CTT	59
16	BCL2	CCT​AGG​CAG​AGC​TGC​GAA​TAA	GTG​TGA​GTG​TGG​CAC​ATG​CA	59
17	CASPASE-3	TCAGGCCTGCCGTGGTA	TCA​TCA​TCA​ACA​CCA​CTG​TCT​GTC​T	59
18	CASPASE-9	GTT​TGA​GGA​CCT​TCG​ACC​AGC​T	CAA​CGT​ACC​AGG​AGC​CAC​TCT​T	59
19	INS	CCCAGCCGCAGCCTTT	GCG​GGT​CTT​GGG​TGT​GTA​GA	59
20	GLUT2	CCT​AGG​CAG​AGC​TGC​GAA​TAA	GTG​TGA​GTG​TGG​CAC​ATG​CA	59
21	PDX1	GCT​GGC​TGT​CAT​GTT​GAA​CTT​G	GGC​GGT​TTT​GGA​ACC​AGA​T	59
22	SYP	CCA​CAG​ACC​CAG​AGA​ACA​TTA​TCA	GTC​ACA​GGG​TCT​CTC​AGC​TCC​TT	59
23	RUNX2	CTG​AAA​AAA​AAA​GGA​GGG​ACT​ATG​G	GCT​CGG​ATC​CCA​AAA​GAA​GTT​T	59
24	OSTERIX	GCCCTCTGCGGGACTCA	GCATCCCCCATGGTTTTG	59
25	RANKL	CTC​AGC​CTT​TTG​CTC​ATC​TCA​CTA	TGG​TAC​CAA​GAG​GAC​AGA​CTC​ACT​T	59
26	SPARC	CTA​CAT​CGG​GCC​TTG​CAA​ATA	GGT​GAC​CAG​GAC​GTT​CTT​GAG	59
27	SCL	GCG​CGT​GGT​TTG​ATT​GTT​TAT	GAT​TCC​TCA​GGG​CCT​GGA​A	59
28	OSCAR	AAC​CCG​CTT​GGA​GAT​TTG​G	GAG​GAC​ACA​TCC​CGG​AAG​AG	59
29	GFAP	GAG​ATC​GCG​ACG​CAG​TAT​GA	ACT​TGG​AGC​GGT​ACC​ACT​CTT​C	59
30	AQP4	CAA​TGA​GAG​CTG​CAC​TCT​GGC	AGG​TTT​CTT​CCG​TTC​CTC​CTT	59
31	S100B	AGA​CCA​GGA​AGG​GGT​GAG​AC	ACT​TGA​ATC​GCA​TGG​GTC​A	59
32	GLUL	GGT​GCT​TGG​ACC​AGC​TAG​AG	GCT​CTG​TCC​GGA​TAG​CTA​CG	59
33	GAPDH	GAC​CTG​ACC​TGC​CGT​CTA​GAA​A	CCT​GCT​TCA​CCA​CCT​TCT​TGA	59

### Induction of HSCs and MSCs to differentiate into specific lineages

#### Induction and characterization of osteocytes

A monolayer of cultured HSCs and MSCs was seeded at a density of 1 × 10^3^ cells/cm^2^ and induced with osteocyte-differentiating medium containing 100 nM dexamethasone, 150 mM ascorbic acid, and 10 mM β-glycerophosphate. The differentiated cell osteocytes were stained with Alizarin Red. Furthermore, the expression levels of Runx2, osterix, RANKL, SPARC, sclerostin, and OSCAR were studied by real-time PCR; the conditions of RT-PCR and primers are mentioned in Table 2. The ALP enzyme activity was studied (Srikanth et al., 2016).

#### Induction and characterization of astrocytes

For astrocyte induction, a monolayer of cultured HSCs and MSCs was seeded at a density of 1 × 10^3^ cells/cm^2^ with 1 μM/mL retinoic acid, 10 ng/mL of FGF, 10 ng/mL of EGF, 20 ng/mL of HCT, and 10 μg/mL of insulin, and the differentiated astrocytes were initially stained with Giemsa. The gene expression of glial fibrillary acidic protein (GFAP), aquaporin 4 (AQP4), S100β, and glutamine synthetase (GLUL) was studied by real-time PCR, and the enzyme assay for glutamine synthetase was performed (Venkatesh et al., 2013).

#### Induction and characterization of beta cells

For beta cell induction, a monolayer of cultured HSCs and MSCs was seeded at a density of 1 × 10^3^ cells/cm^2^ with 50 ng/mL of activin A, 10 ng/mL of epidermal growth factor, 10 ng/mL of fibroblast growth factor, 10 ng/mL of hydrocortisone, 10 mM nicotinamide, 10 ng/mL of triiodo-L-thyronine, and 10 ng/mL of transferrin and was maintained at 5% CO_2_, 37°C, and 95% humidity for 21 days. The morphological changes were recorded using a microscope image processing system (MIPS) (Magnus Analytics, India), and the beta cells were stained for dithizone (DTZ). The gene expression study was performed for INS, glucose transporter 2 (GLUT2), PDX1, and SYP, and the glucokinase enzyme was studied to know the functional ability of differentiated cells (Sunitha et al., 2016b).

#### Viability, proliferation, TRAP, enzyme activity, and quantification of gene expression in differentiated osteocytes, astrocytes, and beta cells

Osteocytes, astrocytes, and beta cells were differentiated *in vitro*, and these differentiated cells were assessed for viability, proliferation, TRAP, enzyme activity, and quantification of gene expression (Kumar P. et al., 2018; Graefe et al., 2019; Ray, 2019; Banerjee and Jagadeesh, 2009; Srikanth et al., 2015; Sunitha et al., 2016a; He et al., 2018; Srikanth et al., 2016; Venkatesh et al., 2013; Sunitha et al., 2016b).

### Statistical analysis

All the experiments were performed three times (n = 3), and all the values were given as the mean ± standard deviation (SD) of the mean. Statistical differences were evaluated using two-way ANOVA with *p*-value < 0.05 considered significant.

## Results

### Isolation and characterization of peripheral blood-derived stem cells

**FIGURE 1:**
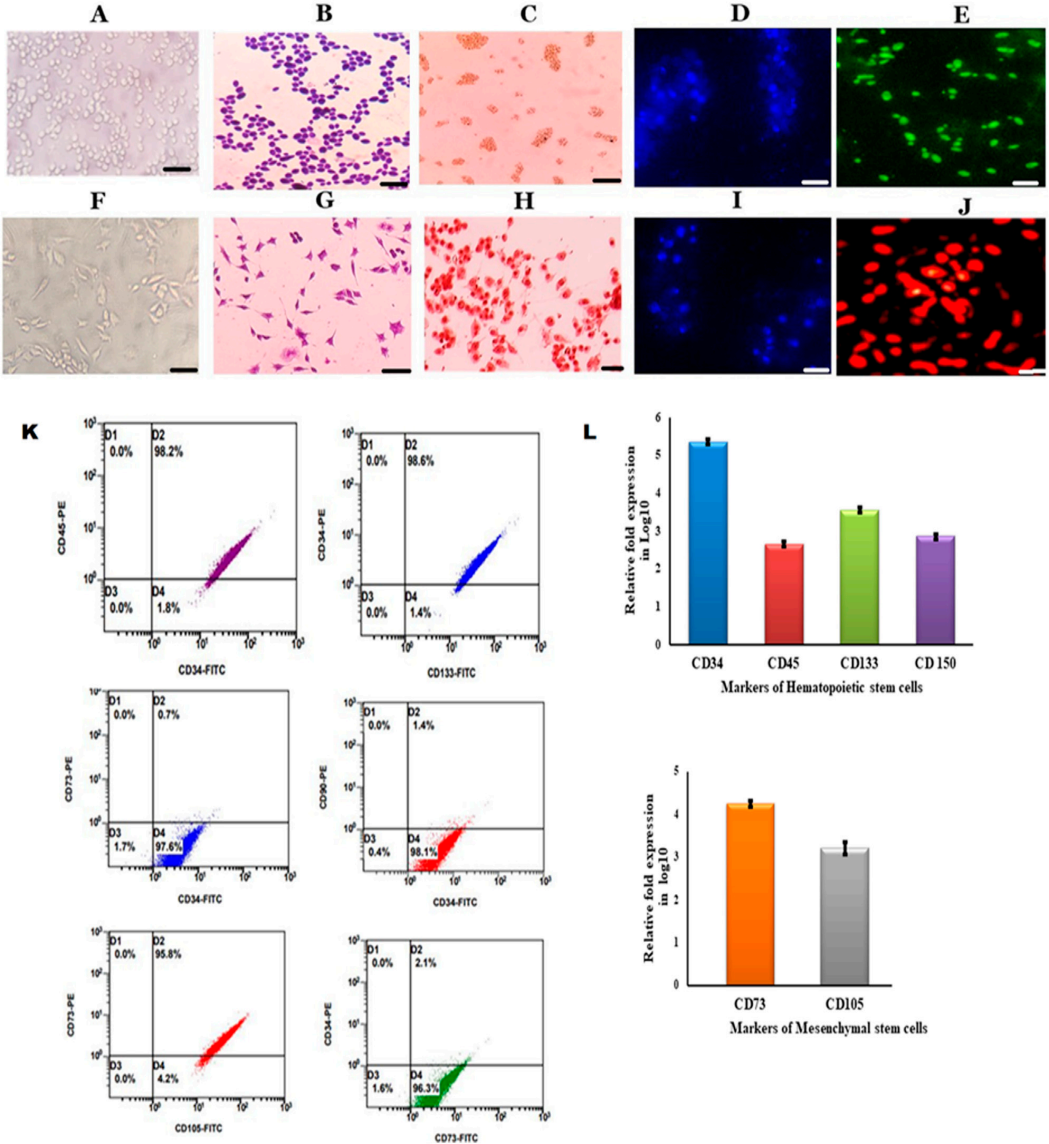
Characterization of HSCs and MSCs isolated from PBSCs **(A)**. Cultured HSCs isolated from PBSCs by the apheresis technique **(B)**. Cultured HSCs were morphologically round with a conspicuous nucleus revealed by Giemsa staining **(C)**. ICC was positive for CD34 anti-human **(D).** DAPI staining for HSCs **(E)**. Immunofluorescence showing growing HSCs positive for the CD34–FITC conjugate **(F)**. Cultured MSCs isolated from PBSCs **(G)**. Cultured MSCs were spindle-shaped with a nucleus **(H)**. ICC was positive for CD73 anti-human **(I)**. DAPI staining for MSCs **(J)**. Immunofluorescence showing growing MSCs positive for the CD73-PE conjugate (scale bar: 50 µm) **(I)**. Flow cytometry analysis of growing HSCs positive for surface markers (CD34, CD45, and CD133) and MSCs positive for surface markers (CD73 and CD105) **(J)**. Growing HSCs express CD34, CD133, CD45, and CD150 on analysis with RT-PCR **(K)**- Flow cytometry analysis of growing HSCs and MSCs showing positive for HSCs surface markers (CD34, CD45 and CD133) and MSCs surface markers (CD73 and CD90) **(L)**. Growing MSCs express CD73 and CD105 on analysis with RT-PCR.

Bone marrow stem cells were mobilized into peripheral blood with 5 μg/kg/day G-CSF for up to 3 consecutive days in a donor, and the mobilized stem cells were separated by using an Rvy kit fitted to an automated blood cell separator system. The obtained cells were cultured in DMEM containing 10% FBS and maintained at 37°C with 5% CO_2_ and 95% humidity. The isolated PBSCs showed the presence of both HSCs and MSCs when PBSCs were subjected to immunocytochemical analysis using CD34 and CD73 monoclonal antibodies. The obtained cells were cultured in DMEM, and these cells were observed for non-adherence and adherence properties. Continuous sub-culturing showed that the isolated PBSCs contained both non-adherent and adhered cells, which were separated and sub-cultured individually with sub-dilutions and characterized for morphology by Giemsa staining. The staining showed that the cells had a conspicuous nucleus and were further characterized for markers by ICC, IFC, and flow cytometry. The results showed that non-adherent cells showing round shaped morphology (Figures A, B, D) were positive for CD34^+^ (Figures 1C, E), and adherent cells (Figure 1F, G, I) were positive for CD73^+^ in immunocytochemistry (Figure 1A). The enumeration of these cells by flow cytometry showed that non-adherent cells showed the presence of CD34^+^, CD45^+^, CD133^+^ (Figure 1L), and CD73^−^, which characterized them as HSCs (Figure 1K). Furthermore, the adherent cells showing spindle shaped morphology (Figures 1F, G, I) that showed CD73^+^, CD105^+^ (Figures 1G, H, L), CD34^−^, and CD133^−^ were characterized as MSCs (Figure 1K). Thus, these results describe the isolated stem cells through the apheresis technique, which were separated as pure HSCs and MSCs. Furthermore, the *in vitro* expansion of these cells showed the continuous expression of specific markers (Srikanth et al., 2015; Sunitha et al., 2016a).

### Proliferation, viability, senescence, quiescence, self-renewal, and bioenergetics in HSCs and MSCs

**FIGURE 2:**
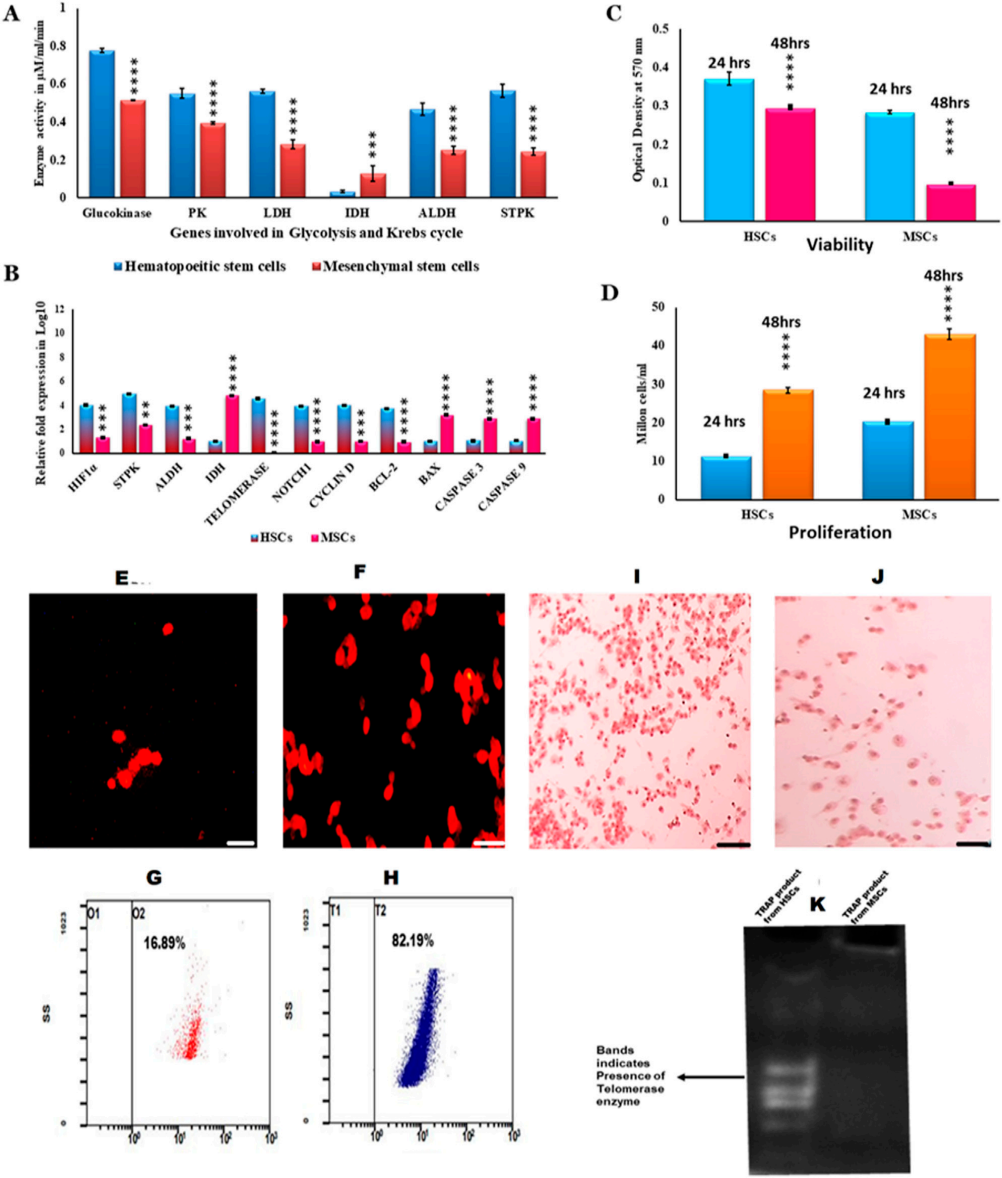
Metabolic and viability characterization of HSCs and MSCs **(A)**. Enzyme activity analysis of glycolytic and TCA-related enzymes in HSCs and MSCs **(B)**. Gene expression analysis of genes involved in the proliferation, viability, and apoptosis of HSCs and MSCs **(C)**. Viability values of HSCs and MSCs obtained by MTT assay at 570 nm **(D)**. Proliferation status of HSCs and MSCs under an *in vitro* condition. **(E,F)**. Ki-67-PE staining of HSCs and MSCs in the immunofluorescence technique **(G,H)**. Flow cytometry analysis of Ki-67 staining in HSCs and MSCs **(I,J)**. Pimonidazole staining of HSCs and MSCs **(K)**. TRAP assay results: lane 1 shows the smear-like band that represents the presence of telomerase activity obtained from HSCs, and lane 2 shows no band, indicating the absence of telomerase. Two-way ANOVA statistical significance: *p ≤ 0.05, **p ≤ 0.01, ***p ≤ 0.001, and ****p ≤ 0.0001.

These characterized HSCs and MSCs were *ex vivo* expanded, and such cells were counted for proliferation at intervals of 24 h and 48 h. HSCs and the viability were assessed by the MTT assay. Furthermore, the proliferation was assessed by fluorescence staining of these cells with Ki-67 staining. The findings indicated that the HSCs were less stained (Figure 2E) with low proliferation rate (Figure 2D), while MSCs were deeply stained (Figure 2F) with high proliferation rate (Figure 2D). Enumeration with flow cytometry indicated 16.89% in HSCs (Figure 2G) and 82.19% in MSCs (Figure 2H). HSCs were highly viable, while viability was poor in MSCs (Figure 2C) (Kumar P. et al., 2018; Graefe et al., 2019).

These results were consistent with those of the TRAP assay, where the findings indicated that HSCs contained prominent telomeres (Figure 2K) and active telomerase enzyme, while they were absent in MSCs (Figure 2K) (Kim and Cho, 2013; Mastrolia et al., 2019) (Figure 2G). These results indicate the continuous self-renewing property of HSCs compared to MSCs in *in vitro* cultures. The metabolic enzyme (GK, PK, LDH, IDH, ALDH, and STPK) activities were estimated in growing HSCs and MSCs. The results indicated that *in vitro* cultured HSCs exhibited high activities of GK, PK, LDH, ALDH, and STPK compared to MSCs, while PDHK activity was higher in HSCs than in MSCs as it is understandable that in HSCs, mitochondria are highly inactive compared to MSCs (Figure 2A). Additionally, the quantitative real-time PCR results indicated very high expression levels of HIF1α, Figure 2B correlated with prominent pimonidazole staining (Figure 2I) STPK, ALDH, telomerase, NOTCH, and BCL2 genes in HSCs compared to MSCs, showing low expression of HIF1 (Figure 2B) concurred with low pimonidazole staining (Figure 2J). Similarly, very high expression levels of cyclin D, BAX, caspase-3, and caspase-9 genes in MSCs were observed compared to HSCs (Figure 2B). These findings indicate the low viability of growing MSCs as opposed to HSCs as growing MSCs undergo rapid apoptosis, which is inhibited in HSCs (Figure 2D) (Srikanth et al., 2015). All these results explain that *in vitro* growing HSCs are more quiescent, with self-renewal property, and utilize anaerobic glycolysis as the major energy source (Srikanth et al., 2015; Sunitha et al., 2016a).

### Differentiation potential of HSCs and MSCs

**FIGURE 3:**
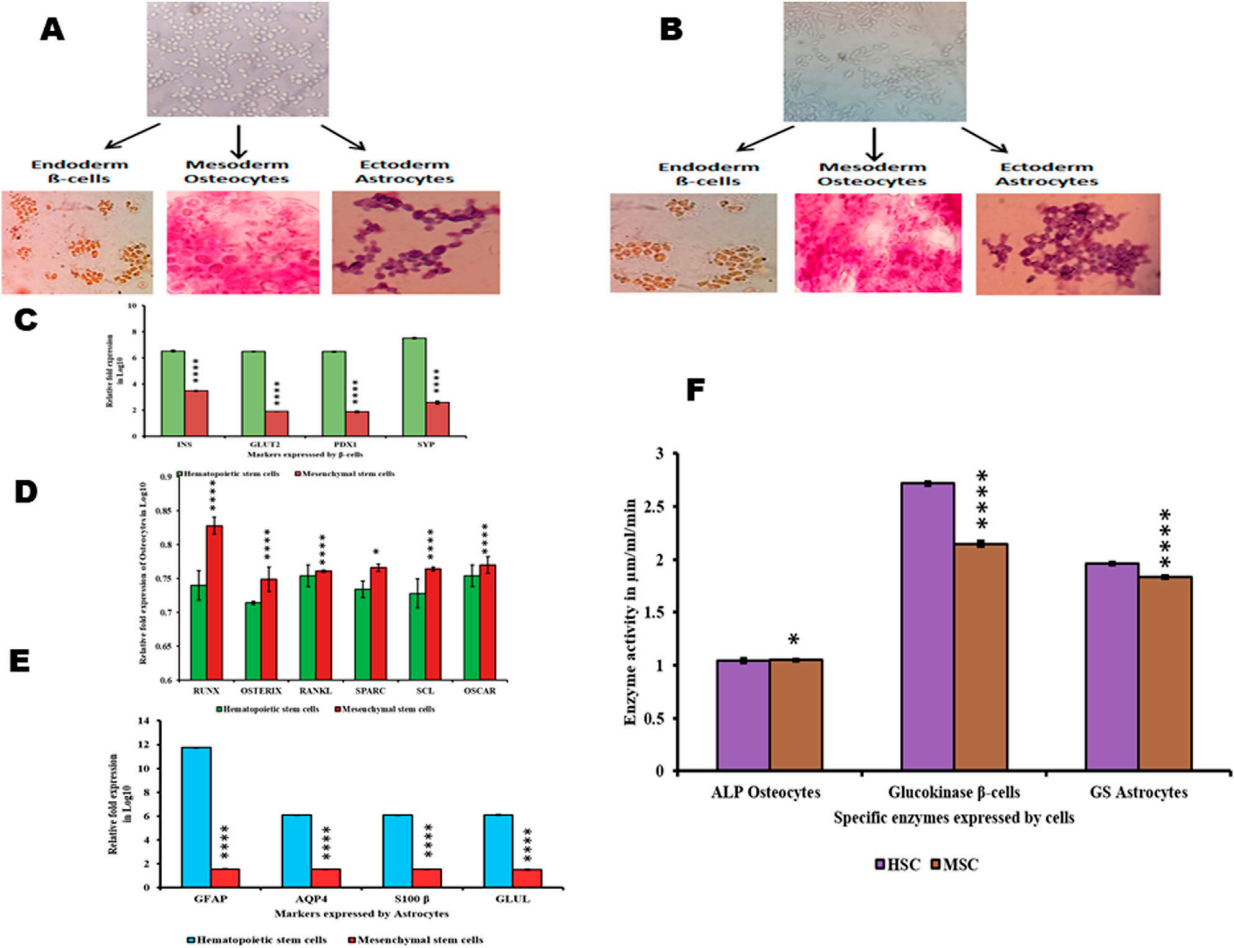
Differentiation of HSCs and MSCs into non-hematopoietic lineages and their characterization **(A,B)**. HSCs and MSCs induced to differentiate into β-cells positive for DTZ staining, osteocytes positive for Alizarin Red staining, and astrocytes were stained with Giemsa **(C)**. Differentiated β-cells expressing INS, GLUT2, PDX1, and SYP **(D)**. Differentiated osteocytes expressing Runx, osterix, RANKL, SPARC, SCL, and OSCAR **(E)**. Differentiated astrocytes expressing GFAP, AQP4, S100β, and GLUL **(F)**. Enzyme activity in differentiated β-cells, osteocytes, and astrocytes. Two-way ANOVA statistical significance: *p ≤ 0.05, **p ≤ 0.01, ***p ≤ 0.001, and ****p ≤ 0.0001.

The ability of HSCs and MSCs to differentiate into different lineages when exposed to lineage-specific media was also evaluated. Both HSCs and MSCs were incubated with beta cell-differentiating media, osteocyte-differentiating media, and astrocyte-differentiating media. We found that both HSCs and MSCs exhibited the ability to differentiate into lineage-specific type of cells (Figures 3A, B). The differentiated beta cells from HSCs and MSCs were positive for DTZ staining (high expression of zinc-bound proteins). Similarly, osteocytes generated from HSCs and MSCs were positive for Alizarin Red stain (indicating calcium deposits). Finally, the stellate morphology of astrocytes (Figures 3A, B) generated from both HSCs and MSCs. Furthermore, in these differentiated cells, the specific enzyme activity was studied as beta cells exhibit high glucokinase activity (Figure 3F) and high expression of INS, GLUT2, PDX1, and SYP genes (Figure 3C). Osteocytes show high alkaline phosphatase activity (Figure 3F) and high expression of Runx2, osterix, RANKL, SPARC, and sclerostin markers, which indicate the formation of osteoblasts. Osteocytes had no osteoclasts as OSCAR expression was very low (Figure 3D). Astrocytes showed high glutamine synthase activity (Figure 3F), while GFAP, AQP4, S100β, and GLUL gene expression was elevated (Figure 3E). These results explain that HSCs and MSCs can differentiate into different lineages if appropriate conditions are provided (Srikanth et al., 2016; Venkatesh et al., 2013; Sunitha et al., 2016b).

### Viability, proliferation, and senescence property of differentiated cells form HSCs and MSCs

**FIGURE 4:**
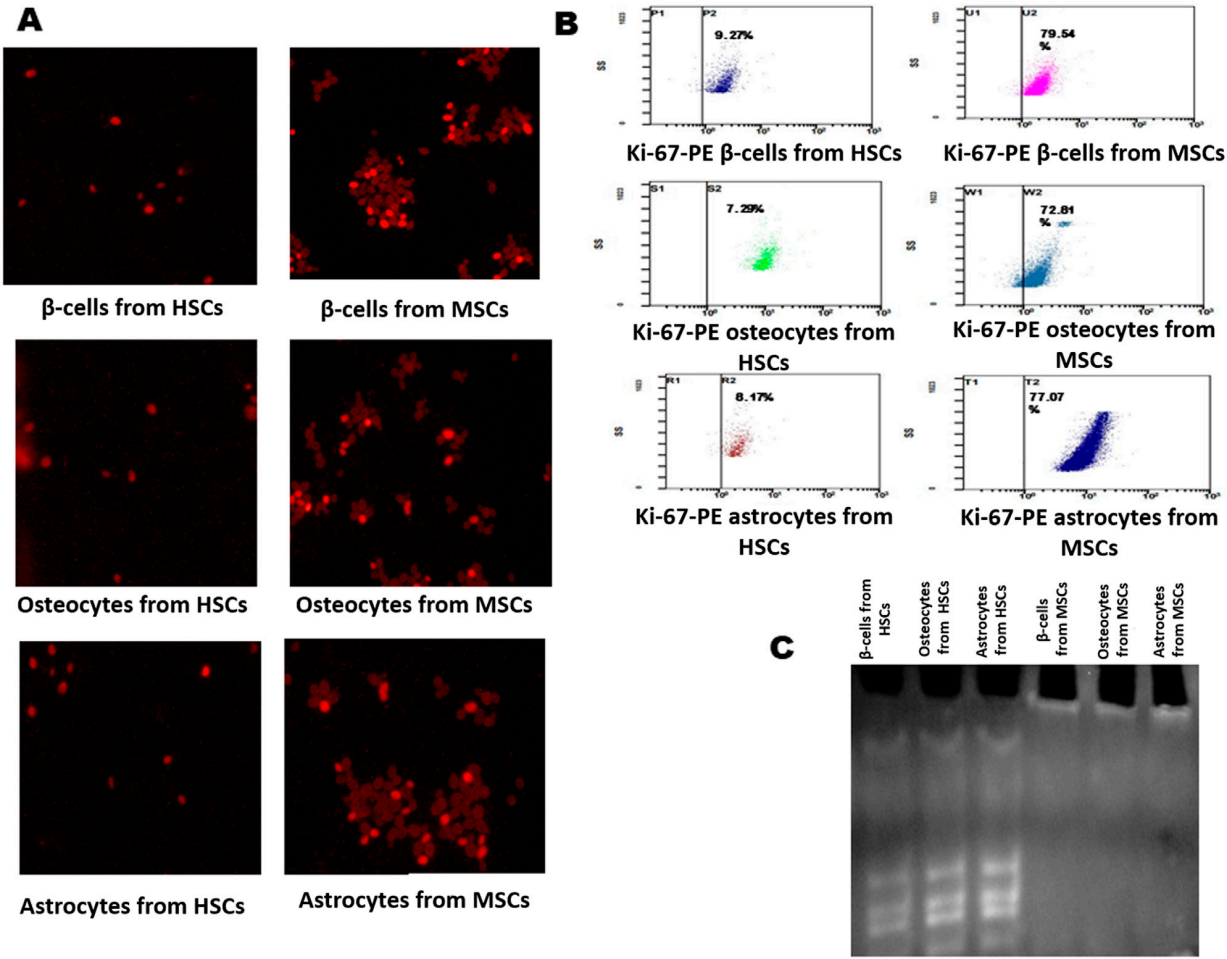
Analysis of Ki-67 proliferation marker and telomerase activity in differentiated cells. **(A)** Immunofluorescence staining of the Ki-67-PE conjugate in differentiated β-cells, osteocytes, and astrocytes. **(B)** Flow cytometry analysis showing the Ki-67 marker in differentiated cells. **(C)** TRAP assay results show the presence and absence of telomerase activity in differentiated cells: lane 1, β-cells; lane 2, osteocytes; and lane 3, astrocytes from HSCs; Lane 4, β-cells; lane 2, osteocytes; and lane 3, astrocytes from MSCs.

The differentiated beta cells, osteocytes, and astrocytes were analyzed for proliferation, and viability was measured by the MTT assay at 570 nm at an interval of 24 h and 48 h. Furthermore, these cells were assessed by fluorescence staining with Ki-67 stain. The findings showed that beta cells differentiated from HSCs were less stained than those differentiated from MSCs, with flow cytometry results indicating 9.27% in HSCs and 79.54% MSCs (Figure 4B). Ki-67 staining showed that osteocytes differentiated from HSCs were less stained and osteocytes differentiated from MSC cells were more stained (Figure 4A), with flow cytometry results indicating 7.29% in HSCs and 72.81% MSCs (Figure 4B). Ki-67 staining showed that astrocytes differentiated from HSCs were less stained and astrocytes differentiated from MSC cells were more stained (Figure 4A), with flow cytometry results indicating 8.17% in HSCs and 77.07% MSCs (Figure 4B). The TRAP assay was performed to study the telomerase enzyme activity in differentiated cells. The results show that cells differentiated from HSC-derived TRAP products were positive with intense bands in 10% polyacrylamide gel, and cells differentiated from MSCs showed no bands, which explains that the telomerase enzyme is absent in cells differentiated from MSCs (Figure 4C). These results explain that although the HSCs and MSCs show differentiation potentiality into three lineages, the proliferated cells maintaining their viability are fewer in cells differentiated from MSCs than those differentiated from HSCs.

### The viability of differentiated osteocytes, astrocytes, and beta cells

**FIGURE 5:**
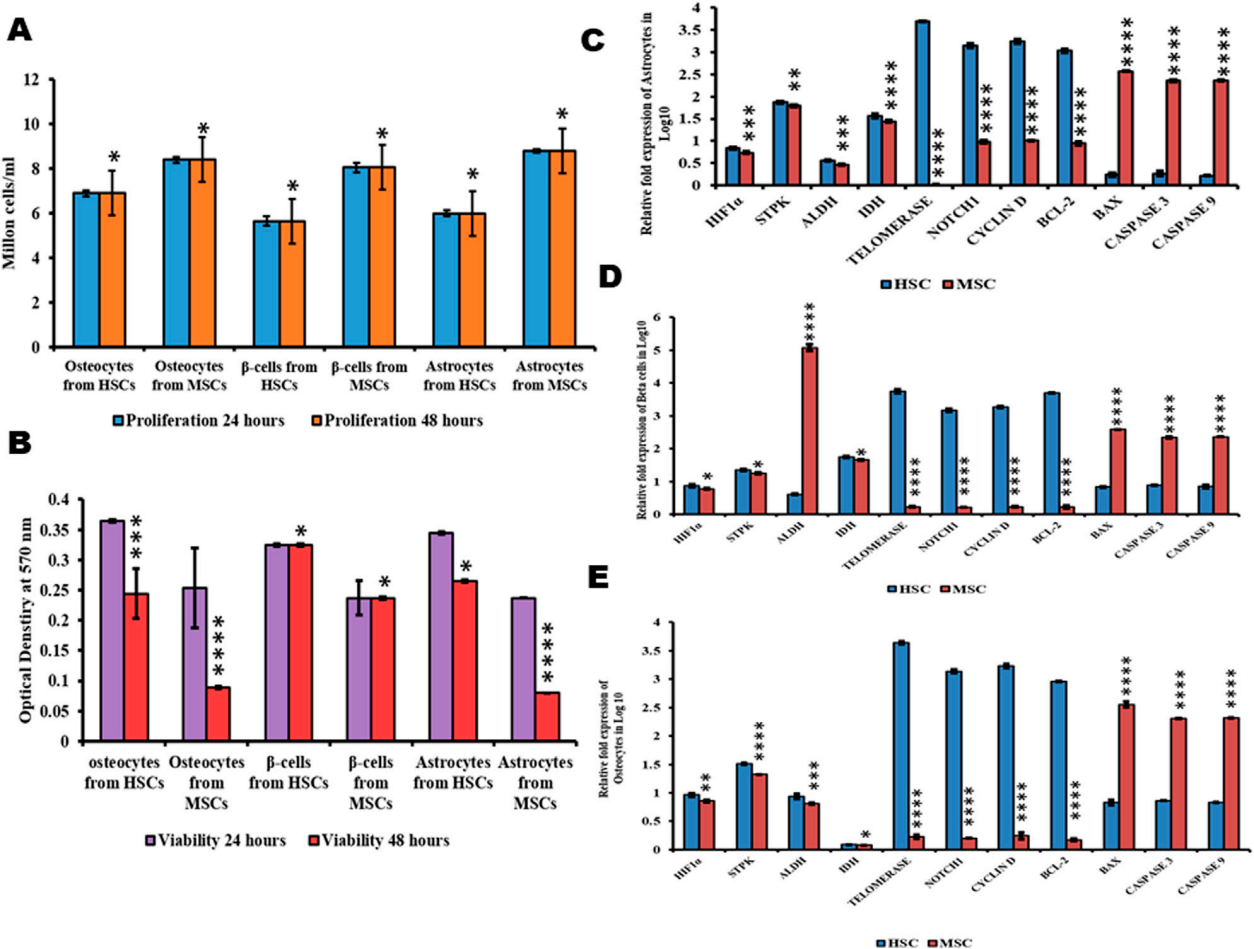
Proliferation, viability, and metabolic status of differentiated cells. **(A)** Proliferation values of differentiated cells. **(B)** Viability values of differentiated cells. **(C–E)** Gene expression analysis of differentiated cells from HSCs and MSCs. Two-way ANOVA statistical significance: *p ≤ 0.05, **p ≤ 0.01, ***p ≤ 0.001, and ****p ≤ 0.0001.

The expression of genes HIF1α, STPK, IDH, ALDH, telomerase, Notch, cyclin D, BCL2, and BAX was studied in beta cells, osteocytes, and astrocytes differentiated from both HSCs and MSCs using quantitative real-time PCR. The results indicated that HIF1α was observed in cells differentiated from both HSCs and MSCs, which was very low compared to undifferentiated cells. Figures 5C–E and correlated with a higher proliferation rate in cells differentiated from MSCs than HSCs (Figure 5A) and the cells generated from HSCs are more viable than cells from MSCs (Figure 5B). The expression of STPK, IDH, and ALDH genes was almost equal in cells differentiated from HSCs and MSCs (Figures 5C–E). Furthermore, the expression level of apoptotic genes BAX, caspase-3, and caspase-9 was low in cells differentiated from HSCs and high in cells differentiated from MSCs, concurring with the results on the expression of telomerase, Notch, and BCL2 genes, which was high in cells differentiated from HSCs compared to cells differentiated from MSCs (Figures 5C–E). These results conclusively explain that the cells differentiated from HSCs were more viable and divided continuously, while cells differentiated from MSCs were less viable and undergo apoptosis.

All these results conclusively explain that HSCs possess the ability to differentiate stably into lineages when incubated in specific differentiating conditions compared to MSCs, where growing MSCs and cells differentiated from MSCs undergo rapid apoptosis, which shows that HSCs are the most suitable cells for the regeneration of injured or damaged tissues.

### Statistical analysis

Using ANOVA with *p*-value < 0.05, the data were considered to be significant with its mean ± standard deviation.

## Discussion

In the present study, we identified the donor and administered G-CSF at a concentration of 5 μg/kg/day for 3 consecutive days, and day 4, peripheral blood stem cells were isolated by the apheresis technique using the Rvy kit (Bernitz et al., 2017). These cells were grown in DMEM for 3 weeks; all the adherent and non-adherent cells were separated. The non-adherent cells exhibited conspicuous presence of CD34, CD133, and CD45 antigens and absence of CD73 and CD105, indicating that all these cells were hematopoietic stem cells (Figures 1K, L), while adherent cells exhibited the distinct expression of CD73 and CD105 and absence of CD34, CD133, and CD45 antigens, indicating that the adherent cells were mesenchymal stem cells (Figures 1C, K, L) (Alhadlaq and Mao, 2004; Sintes et al., 2008; Hao et al., 2016; Szyper-Kravitz et al., 2003). The Ki-67 protein (also known as MKI67) is a cellular marker for proliferation and is present during all active phases of the cell cycle (G1, S, G2, and mitosis) but is less expressed in quiescent cells (G0). The cellular content of the Ki-67 protein markedly increases during cell progression through the S-phase of the cell cycle (Sun and Kaufman, 2018). These plastic-adherent MSCs grew at a faster rate than the slow-growing HSCs. The flow cytometry enumeration results and Ki-67 staining revealed the same (Figures 1E–H). Furthermore, the lower expression of cyclin D in HSCs and increased levels in MSCs validate the slow-growing nature of HSCs, which reaffirms the findings of earlier researchers that the cell cycle is a finely regulated process that enables cellular growth, replication, and differentiation. In particular, the G1-phase appears to function in the mechanism that governs the choice between proliferation and differentiation of stem cell populations. Cyclin D is a member of the cyclin protein family that is involved in regulating cell cycle progression. The synthesis of cyclin D is initiated during G1 and drives the G1/S-phase transition. Cyclin D is expressed in most proliferating cells (Pietras et al., 2011). The prominent expression of the telomerase gene and high positivity in the TRAP assay of HSCs as opposed to MSCs, where no telomerase gene expression was observed (Figures 2B, K), accentuate the long-term stability of HSCs over MSCs (Marx-Blümel et al., 2021).

Researchers have also confirmed the high telomerase expression in HSCs isolated after they were mobilized from G-CSF (Bernitz et al., 2017). HSCs inherently possess the ability of telomerase-dependent telomere lengthening, thus escaping the usual telomere-shortening phenomenon (Morrison et al., 1996). Our results concur with these findings as well (Figure 2K). The findings of immunochemical staining with CD34, CD133, and CD45 (Figure 1C) concur with the results of the quantitative real-time PCR experiment (Figure 1L). As high telomerase activity was noted in the HSCs, we also analyzed CD150 gene expression in HSCs by quantitative real-time PCR; interestingly, the prominent expression of the CD150 gene was observed in the isolated HSCs (Figure 1L), confirming that these HSCs were LT-HSCs that survive for long periods when cultured (Sunitha et al., 2016b; Alhadlaq and Mao, 2004; Sintes et al., 2008; Hao et al., 2016). These HSCs were cultured in DMEM containing 10% FBS, and these non-adherent HSCs grew as floating cells in the medium. Under this condition, these cells expressed CD34, CD133, CD45, and CD150 glycoproteins (Figure 1L). The positive reaction in the TRAP assay indicates that the growing HSCs also showed prominent expression of telomerase (Figure 2K). The main property that the growing HSCs should exhibit is their quiescent nature. Research has shown that CD133 expression pushes the HSCs to go into a quiescent state, suppresses the mTOR pathway, and promotes the expression of genes (HIF1α and Notch1) involved in stemness, thereby helping HSCs in long-term maintenance and self-renewal (Simsek et al., 2010; Catlin et al., 2011). In the present study, we observed the distinct expression of CD133, CD34, Notch, and HIF1α genes in growing HSCs, which helps the HSCs to always be in the quiescent state (Figure 2B).

The metabolic status of HSCs that maintains their quiescent nature was due to anaerobic glycolysis, where majority of the glucose on phosphorylation is converted to ethanol and carbon dioxide. Thus, the formed ethanol is converted to acetaldehyde by the aldehyde dehydrogenase enzyme, and some amount of glucose-6-phosphate undergoes glycolysis. The formed pyruvate is largely converted to lactate, which, in turn, blocks Kreb’s cycle, thereby regulating the redox status, while small amounts of pyruvate converted to acetyl CoA are consumed by histone acetylase, causing the unwinding of DNA, followed by its replication and the proliferation of the HSCs. Furthermore, PDHK prevents pyruvate from being converted to acetyl CoA (Srikanth et al., 2015; Sunitha et al., 2016a; Oburoglu et al., 2014; Ab Kadir et al., 2012). The findings of the present study concur with these findings where in growing HSCs, elevated activities of LDH, ALDH, PK, glucokinase, and serine/threonine protein kinase and lowered activities IDH were observed (Figures 2A, B), which corroborate that in growing HSCs, anaerobic glycolysis prevailed. These results were further strengthened with the increased expression of ALDH, STPK, and HIF1α genes and lowered expression of the IDH gene (Figure 2C). The subtle difference between growing HSCs and MSCs is that reduced expression of HIF1α, ALDH, telomerase, and Notch1 genes was found, and the expression of these genes was elevated in HSCs, accentuating the fast-growing, plastic-adherent, and less quiescent nature of MSCs (Figures 2A, B) (Morganti et al., 2022). The growing MSCs were metabolically very active compared to HSCs as the enhanced expression of the IDH gene and reduced expression of HIF1α, Notch1, STPK, and ALDH genes were found (Figures 2A, B), which explains that glucose is catabolized to generate ATP by allowing the TCA cycle to function, although at a lower rate (Oburoglu et al., 2014; Ab Kadir et al., 2012; Morganti et al., 2022) (Figure 2D).

The HSCs always escape apoptosis because of their self-renewal ability. Here, in HSCs, the constant expression of the BCL2 gene (Figure 2B), which exhibits an anti-apoptotic property by directly blocking cytochrome C release and thereby prevents APAF-1 and caspase-9 activation, makes HSCs escape apoptosis and undergo proliferation (Venkatesh et al., 2013; Hiyama and Hiyama, 2007), whereas the growing MSCs exhibit high expression of apoptotic genes, thereby rapidly undergoing apoptosis (Giacomini et al., 2023). In the present study, we observed that growing MSCs were less viable than HSCs. Furthermore, very high expression of BAX, caspase-3, and caspase-9 genes was found, indicating that they undergo apoptosis (Figure 2B). The growing HSCs can also differentiate into non-hematopoietic cells, as reported by several research groups (Kumar et al., 2017; Kumar P. S. et al., 2018; Ogawa et al., 2013; Wolfien et al., 2020).

When these growing HSCs and MSCs were subjected to an astrocyte-differentiating medium, beta cells of the islets of Langerhans, and osteocyte-differentiating media (Srikanth et al., 2016; Venkatesh et al., 2013; Sunitha et al., 2016b), they successfully differentiated into functional beta cells of the islets of Langerhans, osteocytes, and astrocytes (Figures 3A–F). The functional beta cells of the islets of Langerhans expressed insulin, GLUT2, PDX1, and synaptophysin genes, and differentiated cells stained with DTZ (Figures 3A–C) showed very high glucokinase activity (Figure 3F). Similarly, HSCs and MSCs successfully differentiated into osteocytes by expressing the osteogenic markers, such as osteoblastic markers like RUNX2, osterix, and RANKL; osteocyte markers such as SPARC and sclerostin; and very low expression of OSCAR (Figures 3A, B, D) with prominent expression of alkaline phosphatase (Figure 3F). Furthermore, in the astrocyte-differentiating medium, the growing HSCs and MSCs differentiated into astrocytes expressing GFAP, GLUL, transcription factor S100β, and AQP4 genes (Figures 3A, B, E), with conspicuous GLUL activity in these cells (Figure 3F). When the number of cells differentiated into these cells was observed, MSCs showed better differentiation ability than HSCs; however, the viability of beta cells, astrocytes, and osteocytes differentiated from MSCs was very poor compared to the cells differentiated from HSCs (Figures 5A, B). This is because the cells differentiated from MSCs underwent apoptosis at a very rapid rate compared to HSCs, where the differentiated cells after several passages also remained viable (Figures 5C–E). These observations are consistent with the earlier evidence from other laboratories, which suggests that transplanted MSCs only briefly remain viable in recipients, after which they undergo apoptosis both in the host circulation and in engrafted tissues (Fu et al., 2021; Pang et al., 2021). Furthermore, the complete absence of the telomerase enzyme in MSCs (Figures 1K) could be the reason for the poor viability of these cells. Telomere shortening occurs in most human somatic cells and triggers DNA damage responses that mediate cell cycle arrest or apoptosis, while HSCs can escape this trigger by employing a telomerase-dependent telomere lengthening mechanism in replication (Alenzi et al., 2009). Therefore, differentiated cells from HSCs are highly viable and stable across passages, primarily due to sustained telomerase activity and the lower expression of apoptotic genes such as BAX, caspase-3, and caspase-9. High levels of telomerase prevent telomere shortening, which typically triggers apoptosis or cell cycle arrest, allowing HSC-derived cells to divide continuously. Additionally, elevated expression of pro-survival genes like BCL2 and Notch further supports the enhanced viability of HSC-differentiated cells. Furthermore, the regulation of the cell fate in HSCs is tightly controlled by factors like Notch, which influences differentiation decisions, ensuring that HSCs can give rise to diverse lineages while maintaining their regenerative capacity. These findings suggest that HSCs, with their ability to maintain cell fate determination and prevent apoptosis, could serve as a rich source for regenerating damaged tissues or organs in the human body.

## Data Availability

The raw data supporting the conclusion of this article will be made available by the authors, without undue reservation.
